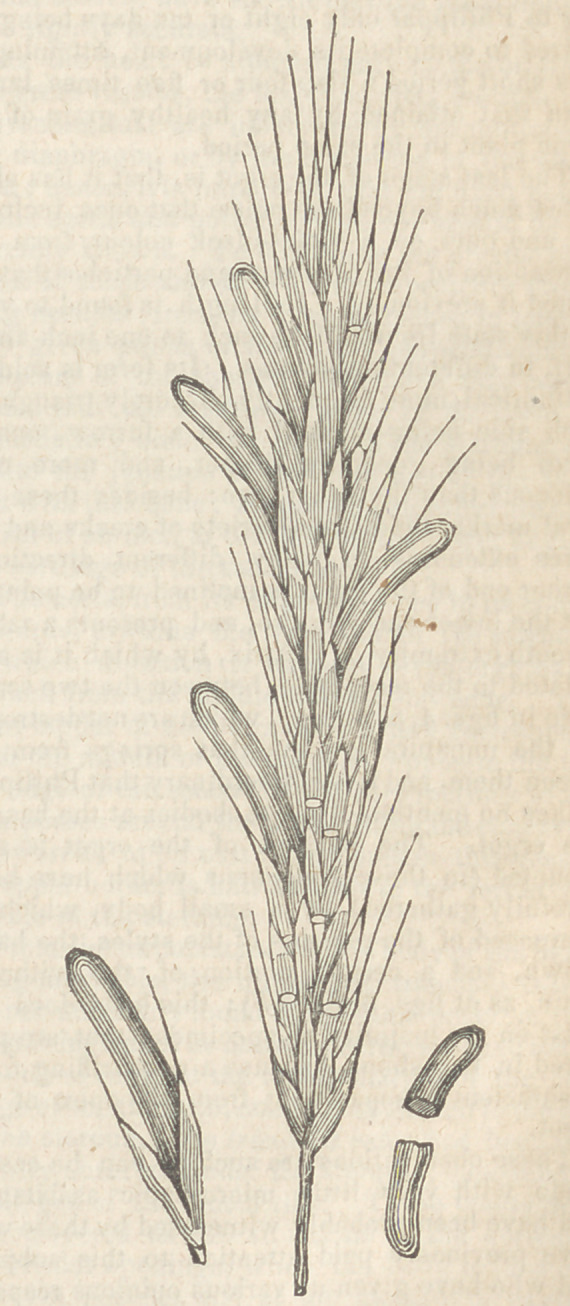# Foreign Summary

**Published:** 1839-08-17

**Authors:** 


### FOREIGN SUMMARY.

*Clinical Observations on Fever.* By Chas. Lendrick, M.D., T.C.D., Queen's Professor of the Practice of Medicine, in the School of Physic in Ireland, &c.

[Continued fom page 516.]

Admitting, as I do, the value of stethoscopism, I am sorry to say that its application in *typhus fever* has, in my opinion, destroyed more lives by the effects of contagion, among medical practitioners and pupils, than it has saved among their patients, by the information it afforded availably for practical purposes. Among the former, I may mention the instance of my lamented friend, Dr. Stack, formerly professor in the School of Physic, and physician to Sir Patrick Dun's Hospital, who, unfortunately for himself, had become imbued with the notion, either that fever was but little contagious, or that he was proof against its influence; and although his long exposure with impunity, while stethoscoping fever patients, seemed almost to countenance such a supposition, his premature death, undoubtedly, and with his own assent, referrible to contagion, afforded a link in the chain of proof as to the danger of the practice, since fully made up by numerous and lamentable instances.

I wish it to be understood that *clinical* lectures are to be considered as commentaries on disease, rather than its description; and that even where the individual cases under hospital treatment are not referred to. I mention this in explanation of the cause of frequent omissions with respect to symptoms and treatment, and which, as well as homeliness of expression, might be avoided in a more methodical consideration of the subject.

The tendency of typhus fever, in the great proportion of cases, is to recovery. The bodily debility—the torpor of the faculties—the stupid, vacant, and yet anxious appearance of the countenance—the furred and coated state of the tongue—and the quickness of the pulse, are observed to increase progressively up to a certain period, when these and the other symptoms undergo an amendment; the time of its occurrence varying from the seventh to the fourteenth day, in the average of instances: yet it is often much later that any thing like a critical change is to be observed, and, in general, the later the period, the less marked and obvious is this change. Whatever may be its amount, the recovery of the patient may usually be dated from it. Sometimes, after an amendment, the disease becomes stationary, and then proceeds to increase, as before the crisis took place. This false crisis is among the worst symptoms of typhus fever, and the instances of recovery after such an occurrence are rare.

It may be laid down almost as an aphorism, that extraordinary occurrences in typhus are bad ones. The very worst symptom in the disease, is a non-agreement of symptoms. For instance, where, with those denoting a severe form of fever, we have a single symptom usually in such cases characterized by morbid alterations, but in the instance before us existing nearly as in the state of health, the prognosis may almost invariably be bad: such, for instance, are complete rationality, or an appetite for solid food, or a regular pulse, or a clean tongue; while the whole course of the disease denotes a case of intense typhus fever. Almost complete inanition, a corpse-like state, with fixed eyes, a hanging jaw, and imperceptible pulse and respiration, were less unfavourable than this, as indicative of the probable result. It is, however, to be borne in mind, that an epidemic often introduces peculiar characters, even in comparatively trivial cases. Thus, of late years, a remarkably slow pulse, sometimes not exceeding forty in a minute, has been an attendant symptom in many mild cases of fever; and yet one which, some time since, would have been justly looked on as the precursor of a fatal termination. The medical practitioner cannot be too little the slave of authority, or too much on the out-look for those changes in disease by which its characters are varied from time to time.

In the great proportion of cases of fever, the physician, bearing in mind its natural tendency to increase up to a certain period and then to decline, will best consult the patient's welfare by almost restricting his treatment to early confinement to bed, (this is the *sine quâ non*,) the use of an apartment large and well ventilated, but neither cold nor containing currents of air, and the administration of tepid diluting drinks, whey, &c., or even such as are cold, (provided they are desired by the patient, and the skin is not disposed to perspiration.) To this treatment ought to be added strict cleanliness, by frequent change of linen, and sponging the skin with tepid or cold vinegar and water, according as it is moist or parched; carefully avoiding, however, permanent exposure to damp, or the risk of catching cold.

There is often much apprehension in the mind of the medical attendant as to the regularity of the bowels. When, however, we recollect that persons in health, and using stimulating food, preserve that health with a single evacuation from the bowels daily, or even less, why should we consider this or more than this necessary, in a disease characterized by debility and collapse, and in which the ordinary means of nutrition are withdrawn? Yet, strange to say, two or three loose evacuations in the day are often considered, by both doctor and nurse, as not only salutary, but indispensable for a safe course of typhus fever. The late Doctor Boyton often remarked, in his clinical lectures at the School of Physic, that he never knew a patient to die with constipated bowels, but numbers of, or at least with, abundant and loose discharges. The general correctness of this observation must be agreed to by every practitioner of experience.

Where, either at the commencement or in the progress of fever, the bowels have been confined for more than a day, with distention or uneasiness of the abdomen, or an increase of febrile symptoms, an evacuation ought, of course, to be obtained. One of the best medicines for this purpose is the oily draught, or from one to two spoonfuls of good castor-oil, floating in a small cup of coffee. If this should be offensive to the stomach, a rhubarb draught* may be exhibited; preceded, where there is reason to suppose an accumulation in the bowels, by Lady Webster's pills, (as described in Paris's Pharmacologia,) or some other aloetic preparation. By such means the contents of the bowels may be removed, without their being irritated by the production of an unusual secretion. The effects of aloes on the rectum are not injurious, when it is exhibited for so short a time, at least to such a degree as to constitute an objection to its use. The administration of enemata, whether in conjunction with the laxatives just mentioned or independently, possesses the great advantage of freeing the bowels for the time, and of doing no more—that is, of evacuating their existing contents, without causing future diarrhœa.

When the bowels have been once emptied, a

* ℞. Infusi Rhei cum duplo Rheo, unciam; Bicarb. Ammoniæ, grana decem; Sulph. Magnesiæ, drachm. =i.—ii. Solve, et fiat haustus. Capiat cum semiunciâ succi limonis in actu effervescenciæ et repetatur horâ tertiâ quâque ad effectum.

single evacuation daily, or that average, (such as two stools on each alternate day,) will be found quite sufficient; and I should be more apprehensive of a considerable variation above than under this standard. It is not improbable that, as some authors have remarked, diarrhœa in the course of fever may be sometimes attributable to neglecting the due evacuation of the bowels at its commencement: yet, for one such case, ten occur where inseparable mischief is done by early purging.

The moderate evacuation of the bowels at a rate not exceeding one discharge daily, with attention to cleanliness, external fomentation, and diluting drinks, constitutes the most successful treatment of fever in the great majority of cases; except, indeed, the occasional use of the effervescing mixture of citrate of ammonia, or of the citrate in the still and neutralized form; there is generally little else to be done, if the head be not much engaged.

Affections of the brain are, however, such dangerous attendants on fever, that the physician ought always to be on the watch for their occurrence. It is no bad rule, in almost every case of typhus, to shave the head. The mere removal of the hair relieves cerebral symptoms; and if headach be present, a cold lotion ought to be applied. Dr. Osborne prefers, for this purpose, cotton wadding to the cloths generally used; and by this, the evaporation of the fluid and the cooling process are much more readily effected. Cold is sometimes overdone; and when the heat of the scalp is found to have diminished to the usual standard, the lotion should be discontinued. A sensation of deep aching pain in the head, is a frequent consequence of lotions being too cold or too long continued. In almost every case where these cold lotions are used, warm fomentations should be applied to the feet, especially if the patient be restless. This application, simple as it is, is a powerful sedative.

I am in the habit of directing the alterative powder used in the Cork Street Fever Hospital, formed of one grain of calomel, and two or three of James's powder. By James's powder I mean the empirical preparation—the imitations in the Pharmacopœia being comparatively worthless. The alterative powder is given every night while head symptoms are threatened, up to the period of convalescence. It forms almost the only exception in my treatment to the principle of waiting for symptoms, instead of anticipating them. I think it is unquestionably beneficial in its action on the brain. It seldom acts on the bowels, or, if it should, this effect can easily be restrained by the addition of an astringent.

Where, however, there is severe pain in the head, with increased determination of blood—where delirium supervenes, or coma is advancing—a more active use of mercury and antimony becomes necessary, and forms, in fact, our most powerful means of saving the patient. The above medicine must, according to the urgency of circumstances, be repeated three or four times in the day; or it may be requisite to give two grains of calomel,* and three of James's powder, every third hour, as in iritis. With such doses, the action of mercury on the bowels must, of course, be restrained; and as opium is, from its influence on the brain, generally contra-indicated, the object may be effected by administering the alterative powder in the form of bolus, with the electuary of catechu (prepared without opium) and kino.

In cerebral cases the constitution is often remarkably repugnant to the influence of mercury and antimony. Two more powerful agents in such cases, we do not, however, possess; and if we succeed in touching the mouth with the former, and producing a slight nausea with the latter, the patient is generally saved. Keeping these objects in view, the doses or their repetition must be regulated by the discretion of the practitioner. In cases of incipient pneumonia, where, with a crepitus near the base of the lung, we have also loss of respiration at the base, and some of the usual symptoms of this disease—where, in short, the existence of pneumonia is unequivocal—this treatment ought to form our principal resource.

Mercury and antimony, however, are to be reserved for the above-mentioned cases, where their administration is necessary, or rather indispensable. They are both liable to the serious objection of producing irritation in the stomach and intestines. I never concurred in the hypothesis of gastro-enteritis being the essence of typhus fever; but undoubtedly it is such a frequent attendant, and so dangerous and unmanageable a one, that we cannot be too careful in avoiding any mode of treatment, not otherwise indispensable, which might favour its production; more especially if any of the symptoms which usually announce its invasion, such as nausea, diarrhœa, pain on pressing the epigastrium, or redness of the tongue, should have made their appearance.† For this reason, where antimonials are necessary, I generally prefer James's powder to the tartarized antimony, which produces a more irritating effect on the stomach and bowels.

General blood-letting is rarely practised in Dublin in typhus fever unconnected with local inflammation, the erroneous pathological notions which led to its adoption having long since disappeared here. In cases of undue determination to the head, local bleeding is the mode we usually have recourse to. The medical attendant ought to bear in mind, however, that a florid hue of the countenance, throbbing of the vessels, and some difficulty of respiration, are often merely symptoms of that general excitement which precedes a critical change, or at least an amendment in

* Dr. Law's mode of affecting the mouth by minute doses of calomel, such as a quarter of a grain every hour, is comparatively free from the objection of irritating the bowels.

† Where these symptoms are not present, the combination of tartar emetic and opium, administered according to the directions of Dr. Graves, affords a useful remedy in the sthenic forms of delirium, as referred to in a subsequent part of these observations.

fever; and that depletion then is more likely to be injurious than beneficial. This remark was made long since by our fever hospital physicians.

Where pain in the head exists, attended by determination of blood, there can rarely be any injurious effect from the application of a few leeches. On the contrary, the patient will generally find the symptoms relieved and restlessness diminished; and it may, in my opinion, be laid down as a rule in the treatment of fever and most other diseases, that relief from suffering of any kind is always desirable as a means of cure. With this view, where the patient suffers from want of sleep, amendment is accelerated by administering at night a pill of three grains of good extract of hyoscyamus. The use of a larger dose, or of any preparation of opium, is, under such circumstances, a more questionable matter, and will be considered hereafter.

In order to avoid unnecessary annoyance, it will generally be the better way to apply the leeches (six or eight in number) to one temple, to cause the patient to lie on the opposite side, and to place a few folds of clean linen over the leech-bites. When the cloth is saturated with blood it may be removed, and the temples lightly bound up: thus the annoyance of steeping is avoided. This is Dr. Osborne's practice. A patient should never be allowed to lie on a bleeding surface. Under particular circumstances, an unseen bleeding may constitute a dangerous hæmorrhage.

The loss of a few ounces of blood thus gradually oozing from the temples, much relieves cephalic symptoms, without exhausting the general strength. On the principle, therefore, of not doing more in fever than is necessary, this mode of abstracting blood ought always to precede more energetic measures of the kind. If, however, the symptoms should continue unrelieved, or delirium supervene, a more active abstraction of blood is requisite. No mode is more powerful, or, as far as an efficient removal of blood without constitutional exhaustion is concerned, more advantageous, than temporal arteriotomy. Unfortunately, however, the other consequences are sometimes such as to render its adoption in private practice, unless in a case of great urgency, far from desirable. The object of abstracting arterial blood, and in almost any quantity required, can be attained by cupping the temples. For this purpose, good and peculiar apparatus, with dexterity in its use, are indispensable. Adequate directions for the performance of the operation are given by Mr. Hills, in his little treatise on Cupping. The practice of cupping the temple was introduced into our clinical hospital on the recommendation of Dr. Swan, during his pupilage, and who displayed great skill in its performance.

Should pain in the head, with determination of blood, continue unabated after cupping the temple, the antimonial and mercurial measures aforementioned should be had recourse to; and in the meantime, if the cupping should have been unsuccessful, blood ought to be abstracted directly from the temporal artery, especially if the pressure of the cupping-glass is irksome. The best way to avoid the bad consequences of arteriotomy is to make the incision at least an inch from the zygoma, and directly in the course of the artery—neither across nor obliquely. Secondary hæmorrhage and aneurism are thus less likely to occur, or other parts to be wounded, when a puncture is made, on the failure of the incision to open the vessel. A good lancet is the best instrument for the whole operation; and it ought to be held between two fingers and the thumb, and not merely between the forefinger and thumb, as in ordinary venesection. If the patient be not refractory, the best mode of stopping the bleeding is to cause an assistant to place his finger on the wound for some time till the disposition to bleed cease, and then to apply sticking plaster, a compress, and a triangular handkerchief as a bandage, lightly. If the patient be violent, the bleeding considerable, or professional assistance not likely to be at hand, the better way is to raise the artery on a tenaculum or needle, to cut it across, and then to apply the bandage. In every case the patient ought to lie on the opposite side, so that the wounded temple may be in view, and any disposition to hæmorrhage at once discovered. Lives have been lost from the neglect of this precaution. It would be well, indeed, that all attendants on the sick, and indeed people in general, were aware that the most serious hæmorrhage, even that from a considerable artery, may be kept in check for any required time, and till surgical assistance can be procured, by merely placing a bit of rag over the bleeding point or surface, and gently pressing or rather keeping it in contact by means of one or more fingers, another person relieving the operator if fatigued. It is only necessary that the pressure be unceasing, and of course that the part should not be uncovered even for an instant. This rule applies to all external hæmorrhage, whether from surgical operations, wounds, leech-bites, varicose veins, &c.

Where a patient is violently delirious and intolerant of restraint, I quite concur in the opinion generally acted on here, of allowing every degree of liberty compatible with safety. Cases have occurred of a patient rushing, under such circumstances, into the open air, not only without injury, but with advantage; and I should have no objection to permit him, when violently bent on accomplishing his purpose, to go out of the house in fine weather, if properly clothed and attended. It is generally admitted at the present day, that the best and safest way to remove maniacal excitement, is to permit the ebullition to take place, and thus to pass away. In yielding to the wishes of the patient by affording liberty, precautions as to safety must, of course, be adopted, and sufficient attendance provided.

In these cases of violent excitement, the cold *douche*, by means of a common cullender on the head, is highly advantageous, and still more so if the patient can be persuaded to sit at the same time in a *semicupium* of warm water, and which is readily formed of a common washing tub.

After an application of this kind, and an indulgence of liberty, sleep is often procured, although absent before. Should it be otherwise, three grains of extract of hyoscyamus should be administered. If the patient continue to be restless, an eighth of a grain of acetate of morphine, with half an ounce of Mindererus's spirit, and six drachms of camphor mixture, ought to be given every four hours till sleep is obtained, and which, in such cases, is generally followed by convalescence. The practitioner should always, however, bear in mind the great susceptibility to the influence of narcotics which exists in typhus fever. In a case* lately in the clinical hospital, narcotism was produced by a dose not equivalent to more than half a grain of opium. A gentleman labouring under typhus, with maniacal excitement, whom I attended many years ago with the late Dr. Cheyne, fell asleep, and convalescence ensued, on the administration of two grains of Dover's powder. Where, however, fever is complicated with delirium tremens, a more active use of opium may be requisite; but even here we ought to feel our way with graduated doses of morphine,† progressively increasing.

It is a good general rule to abstain from the use of blisters during the presence of febrile excitement. It is, indeed, to the stage of coma, or the approach of collapse, that these applications appropriately belong. At a much earlier period, however, of typhus combined with phrenitis, we may have recourse to the tartar emetic ointment, by means of which, applied to the scalp, (as the surgeon of our Foundling Hospital, Mr. Creighton, long since remarked,) the specific antimonial influence, so beneficial in such cases, is introduced into the constitution, while a powerful external stimulus is established.

It would be to go over nearly a course of lectures on the practice of medicine, to describe all the forms in which local inflammation combines with typhus fever. In short, any of the phlegmasiæ of the viscera may attend on typhus, and that in two modes:—1st. Where the predisposition to such local disease is strong, or the influence of the accessory causes of fever considerable, the inflammatory symptoms are generally simultaneous with, or, indeed, rather precede the others; and a distinction of the case from one of simple organic inflammation can only be made by observing some of the lurking symptoms of the typhoid affection, which is now masked by those of inflammation, such as the peculiar expression of countenance, petechiæ on the skin, or the (for mere inflammatory disease) unusual loading of the tongue. These distinctions are of the last importance, as, if depletion were had recourse to, to the full extent that inflammatory symptoms might seem to require, the patient would be irretrievably sunk. It is therefore a good rule to practise blood-letting, on account of inflammatory diseases, cautiously, when typhus is epidemic, or where the patient has been exposed to contagion, even though typhoid symptoms have not yet appeared. 2dly. Symptoms of inflammatory disease do not take place till the secondary period of typhus, or that of reaction or excitement. This reaction is, in typhus fever, often scarcely observable, or it assumes the form of mere congestion; but in some cases it is fully developed, and constitutes local inflammation. A diagnosis from mere inflammatory disease, is obviously less difficult in this case than in the former, on account of the previous typhoid symptoms.

The following practical rules may, I think, be advantageously adopted in the treatment of organic inflammation, combined with typhus fever:—

1st. To practise blood-letting, or other depletory measures, only to the amount indispensably necessary for the relief of the inflammatory symptoms.

2dly. To remove blood, as far as practicable, at an early period of the case, when its abstraction is most beneficial to the inflammatory and least injurious to the typhoid symptoms.

3dly. To prefer local bleeding, where sufficient for the purpose, to venesection, as producing less exhaustion of the constitutional powers.

4thly. To adopt, where admissible, those measures which are usually found to be auxiliary to blood-letting in inflammatory diseases—such as antimony, where there is no irritation of the stomach and bowels; or digitalis, where there is no tendency to coma. Thus an unnecessary loss of blood for antiphlogistic purposes may be avoided.

In the ensuing observations as to the use of wine and other stimulants in typhus fever, the cautions as to their administration apply with redoubled force where actual inflammation is combined with typhus, as we have then more serious results than the production of mere excitement to apprehend from their over administration. These are the really difficult cases of fever to manage, and which task the utmost skill, vigilance and caution, on the part of the practitioner, whether in the contemporary or alternate use of depletory and stimulant measures. Let him, however, recollect the golden rule, to do with respect to both what is necessary, and no more, and he will have no cause to blame himself for the result, whatever it may be. Sometimes it is necessary to remove blood by local means from the head, or to practise venesection on account of pulmonary inflammation, and also to administer wine to support the general strength the same day.

Coma is frequently the state whereby the excitement of fever changes to collapse. We accordingly have comatose symptoms present in both these stages of the disease, and its treatment partaking of that of both. Thus, while we continue the use of mercury and antimony, and even of local blood-letting from the head, it may

* It is unnecessary to specify cases where the details are unknown to the reader.

† See last note in page 525.

be also necessary to apply blisters to the calves of the legs, and mustard cataplasms to the feet, to excite the dormant powers of life, while we endeavor to obtain a derivation from the cerebral circulation. This treatment is especially requisite in what are termed the congestive or apoplectic forms of typhus, when the period of collapse, which often takes place early, has arrived.

The collapse of typhus fever occurs in two modes—suddenly, and often preceded by high excitement or coma; or gradually, as it were by the powers of nature being worn out. The first is the more dangerous, not only in itself, but also as rendering the stimulating treatment, which is indispensable, more precarious. The supervention of gradual collapse may be warded off by meeting the symptoms of debility as they become progressive, by means of light nourishment—such as panada, weak chicken broth, caudle, &c. By thus administering nourishment and a little wine, on the first accession of symptoms of increasing debility, I have often, I am certain, succeeded in materially lessening the duration of the disease and the amount of the collapse. Such cases are, however, comparatively of easy treatment: I mean those where we have mere debility to cope with, and there is little difficulty in regulating the amount of stimulus. It is where cerebral congestion, or the dregs of previous excitement, or of inflammation itself, are co-existent with an increasing debility threatening complete collapse, that the utmost circumspection is necessary. Support of some kind is obviously indispensable, and yet, if it be at all overdone, we make matters worse than before.

Nothing proves more, in such cases, the susceptibility of the constitution to undue stimulus than the return of febrile excitement, or even a very small portion of any animal preparation or solid food being administered. The injurious consequences of these is greater, as being more permanent than the effects of wine itself. It is the general practice in the clinical hospital of the School of Physic, on the supervention of collapse suddenly, or where much excitement has preceded it, to exhibit what is termed the cardiac mixture, (a combination of camphor, Hoffman's ether, and ammonia,) and to resort to more active stimulus only on the failure of this to preserve the strength of the system. Should excitement be produced by the cardiac mixture, the physician learns that, *à fortiori*, stimulus by means of wine and nourishment, would have proved prejudicial.

Musk is generally considered as especially applicable to the spasmodic symptoms of fever, such as subsultus tendinum, rigid contraction of the muscles, tendency to convulsive contractions, &c. I think its beneficial effects are almost equally remarkable in comatose cases. An experienced nurse once remarked to me, that "it cleared the head;" and, undoubtedly, we often observe a diminution of stupor, and a return of consciousness, and of a susceptibility to external impressions, consequent on its use. Musk must, however, be of the very best quality. The administration of an inferior article, (generally known from the absence of the peculiar odour in the patient's apartment,) is loss of time, money, and life. I generally prefer the exhibition of musk in the form of bolus, from fifteen grains to half a drachm daily, mixed with conserve of roses and syrup—a dose of the cardiac mixture being given at the same time.

Wine, says Dr. Buchan, in his Domestic Medicine, is worth all the other cordial medicines put together; and the Doctor says truly: as also in his subsequent remark, that in order to bear out his encomium, the wine must be "sound and good." For this reason I think that wherever economy is an object, Cape Madeira is entitled to a decided preference, inasmuch as the best Cape wine is cheaper than the worst port or sherry. Therefore, if price is to be considered, let Cape Madeira, of the best quality, be preferred as the soundest, although, perhaps, not the most agreeable wine.* In the upper ranks of life, the selection of the quality of wine is generally regulated by the taste of the patient. One wine will often agree when another does not. All may be brought to the same strength by more or less dilution with water. In general, when we try wine first, or where the patient is a female, or under puberty, claret, sauterne, or the light Rhenish wines (if there be no tendency to diarrhœa) are to be preferred. Persons of luxurious habits are often more benefitted by Madeira than any other wine, especially if the weaker kinds have failed as a stimulus. Champagne (if genuine) is especially adapted to what are called putrid cases, with great thirst, black tongue, fœtid discharges, and livid petechiæ. Good port is an excellent average wine. It ought to be diluted with water at first.

It is however, of more importance to determine the time for having recourse to wine, and the quantity to be used, than its quality, (provided always that Dr. Buchan's conditions be complied with.) In private practice, wine is given in fever and other cases as a restorative on convalescence taking place, just as it is used as a luxury during health. What, however, I have now to do with, is the *necessary* use of wine as a medicine, during the collapse of fever, or on its approach.

I know of no means of judging, *à priori*, as to the eligibility of using wine. This point, as

* For this observation I am indebted to the late Dr. Robert Perceval—a physician without a superior among his contemporaries or successors, whether we consider his professional and scientific attainments, or his acquaintance with elegant literature. Many years since, he predicted the revolutions that have since taken place in medicine, and boldly raised his voice against the delusive theories of the day, and the equally delusive theories which were destined for a time to supersede them. He considered no subject, however apparently trivial, as undeserving the researches of his powerful mind, provided it could add even an atom to the mass of useful medical knowledge, or contribute to the welfare or comfort of his fellow creatures. *Vivit post funera virtus.*

well as that of increasing or diminishing the quantity, can be learned only from the effect—that is, by *experiment*. In a doubtful case, I have always reasoned with myself, that a wine glassful of claret and water could scarcely do harm, even where marked excitement was present, while its effects would enable me to judge of the susceptibility of the system to its influence, and the expediency of increasing the quantity, or *vice versa*. Generally speaking, cases with a moist skin bear wine better than those where the skin is parched and dry; and slow and gradual debility at a late period, better than where this occurrence is abrupt and early. To such, and all other rules, there are, however, numerous exceptions.

It is obvious that a man of luxurious habits, accustomed to the use of wine, must require a much larger quantity of it to produce a given effect, than a lady, who, perhaps, did not drink a glass for his bottle. In the average of cases, from four to eight ounces of port wine is the quantity found to be most beneficial at Sir Patrick Dun's hospital. It sometimes happens, however, that in a case of collapse, amounting almost to inanition and apparent extinction of life, the vital powers are preserved, and recovery effected, by the use of a bottle or more of wine daily. Yet where such treatment has proved successful, it has always, as far as I have observed, been the result of a gradual increase of wine up to the quantity aforesaid—a feeling of the way—and not by a hap-hazard use of it, which generally fails, or proves injurious.

Whether circumstances justify the use of a single glass of wine, or of one or two bottles daily, the same rules may be applied. 1st. If wine is agreeing, and the patient is improving, "let well enough alone." The quantity of wine ought on no account to be increased—nay, if it be already large, it is to be borne in mind that as the patient improves he will become more and more susceptible of its stimulus, and therefore the medical attendant ought to be rather beforehand with the symptoms of excitement, and to diminish the large quantity of wine before they appear. 2dly. If the symptoms indicate that wine is disagreeing, the quantity ought to be reduced, the quality of the stimulus changed, or it ought to be laid aside, according to the degree or duration of such disagreement. 3dly. If wine is producing no effect whatever, and the symptoms of debility are progressive, as before its administration, the quantity ought to be cautiously increased.

When wine is agreeing with a patient in fever, we observe an amendment in the symptoms, which seems to the observer as if it were the spontaneous effort of nature, and not the result of the influence of a stimulus. The patient becomes stronger, more conscious, and less restless; the pulse is slower and firmer, and the tongue cleaner; even the skin, if previously parched, becomes cooler. But there is nothing resembling the stimulating action of wine on a healthy person—no acceleration of the pulse, or flushing of the countenance. Wherever such take place, or where the favourable symptoms, consequent on the use of wine, just mentioned, are observed to be of but temporary duration, we may rest assured that we are verging on the unfavourable influence of the remedy, and that nothing will be gained, and probably something lost, by continuing to administer it in its present quantity. If the pulse be considerably or permanently quickened, or the patient hot or restless—if there be delirium, headach, oppression, or increased quickness of breathing, or the symptoms of approaching or recurring local inflammation, the morbid influence of wine is obvious.

It sometimes happens that by varying the quality, as well as the quantity of the stimulus, it is found to agree, although formerly acting injuriously. Thus white wine will succeed on the failure of red, and *vice versa*. Malt liquor is sometimes advantageously substituted for wine, and I have seen cases where the brisk, home-made wines agreed extremely well. As a general rule, however, no stimulus is equal to good foreign wine. The cardiac mixture which preceded its use, ought to be continued along with it.

The cases which bear porter or ale better than wine, are those where, with great exhaustion or emaciation, there is also much excitement, or a tendency to pulmonary irritation. Malt liquor, as being more nutritive, and less stimulating, is often preferable in such cases. It is also well adapted, in conjunction with wine, where there are bed sores, erysipelas, or gangrene, conjoined with fever. It is unnecessary to add, that porter or ale ought to be of the purest quality. Sydenham was a great advocate for small beer. In some cases, where great determination to the head, or the co-existence of pneumonia, bronchitis, or pulmonary irritation, rendered the use of wine inadmissible as a means of withstanding the advance of collapse, I have allowed the patient a quart or more of light table beer, in the course of the day. Thirst was quenched, and the strength preserved by this treatment, till the typhoid disease underwent a favourable change, and this without any aggravation of the pulmonary or cerebral symptoms. The rules for the administration of malt liquor are similar to those for the use of wine, as to the increase, continuance, or diminution. In cases of great debility, it is often preferable to administer the large quantity of stimulus that the patient requires in different forms. For instance, instead of allowing a bottle of wine daily, to give half a bottle of wine and a bottle of ale.

Spirituous liquors are liable to the objections applicable to wine, without possessing its advantages—that is, they rather produce temporary excitement than permanent strength. In cases of great debility, however, a spoonful of brandy may be added to the patient's caudle, panada, or arrow-root, which is often lighter thus, than by the addition of wine. A more active administration of spirituous liquors is only admissible in cases where the patient has been habituated to their excessive use—where wine acts like water, and spirits like wine. Fresh barm is a great favourite with the Dublin physicians. It certainly agrees best with what are called the putrid cases. Two or three spoonsful may be added to a pint of ale or beer. Some practitioners administer ale-wort, or infusion of malt boiled with hops, during the process of fermentation with yeast. Care is requisite in its preparation. The infusion is to be made by percolating water through the malt. Directions for this purpose are given in Mr. Donovan's Treatise on Brewing, in Lardner's Cyclopœdia—London, 1837, vol. i., p. 205. The directions must be strictly followed, or the requisite chemical changes will not take place. A quart of wort, prepared by means of any small straining apparatus, will require about two pounds of ground malt, a quarter of an ounce of hops, and a table-spoonful of barm. The water required will be nearly two quarts; the first half being added nearly, and the second altogether boiling, for the reasons stated by Mr. Donovan. A decoction of malt and hops made in this way, but in larger quantity, and without the addition of yeast, inspissated by heat to the consistence of molasses, is an excellent demulcent during irritation of the bronchiæ, from whatever cause. A tea-spoonful is to be taken occasionally.

In cases of great debility, especially if connected with local gangrene, and where other nutriment fails in supporting the strength, the patient may be nourished by means of beef gravy or osmazone, given in spoonfuls from time to time. A pound of very fresh, juicy, and lean beef, will afford about a quarter of a pint. The raw meat is to be sliced, scored on the surface, sprinkled with a little salt, and put into a close vessel with two spoonsful of water, (not more,) over a slow fire, for from twenty minutes to half an hour. The juice exudes under the influence of the heat, and is ready for use.

It is an aphorism in fever, that the patient is never to be given up. Cases have repeatedly occurred, where the physicians have taken their leave during what was supposed to be the death-struggle—nay, life has been supposed to be extinct, and the body put into the coffin, and yet the patients are at present alive and well. The favourable turn in the disease, often scarcely perceptible at first, is, therefore, always a matter of reasonable hope, even in the apparently worst cases. Every mode is to be had recourse to, to prevent the extinction of the vital spark; wine—burned brandy (in such cases)—beef gravy—musk—cardiac mixtures—and counter-stimulus, by means of blisters to the calves of the legs, the upper arms, and the nape of the neck. The sores produced by the last mentioned applications often, however, prove troublesome subsequently, and require careful treatment. When we consider, indeed, the slender link which connects the patient with existence in this world, it must be obvious that the slightest variation in the performance of professional duty must often strike the balance between life and death, and that even the slightest aggravation of the danger ought to be withstood by adequate precautions. During the period of utter helplessness and unconsciousness, the patient is exposed to additional peril from three causes; inattention to cleanliness—stripping and sloughing—and retention of urine. To these I shall next direct my observations.—*Lon. Med. Gaz.*

---

*Observations on the Anatomical and Physiological nature of the Ergot of Rye and some other Grasses.* By Edwin J. Quekett, Esq. F. L. S., &c. Lecturer on Botany at the London Hospital and Aldersgate School of Medicine.

[Abridged from a Paper read before the Linnean Society, November 4, 1838.]

The investigation of this peculiar formation has often occupied the attention of both English and foreign botanists, with the view of determining its nature and origin; yet notwithstanding the mystery belonging to it has not been completely removed, the observations of some of the latter authorities have gone far towards our viewing this substance in a clearer light, especially those of Dr. Phœbus, in the *Deutschlands kryptogamische Giftgewächse*, and of Philippar, in his "*Traité Organographique et Physiologico-agricole sur l'Ergot, &c., dans les Cereales;*" from both of whom we learn much interesting matter, and also the history and former hypotheses of the ergot, which here will be omitted, for the sake of shortening this communication.

After many attempts at the examination of the ergot of rye in the state it is generally found in the shops, I could never succeed in finding any thing from such specimens, respecting its structure, that served to identify it satisfactorily with any other vegetable production; consequently it has long been my wish to obtain recent specimens of the rye, or any other ergotized grass, in order to trace the growth of the ergot from its first commencement, which I have this year been enabled to do; and one grass in particular, the *Elymus sabulosis*, a plant much larger than the rye, has afforded an excellent illustration of the growth and development of this anomalous formation.

In order to trace the ergot through its several phases, it is necessary to become acquainted with the normal size and characteristics of the grain of the several grasses, in its various stages, whilst healthy, and also the same conditions of its appendages, which may be probably understood from the figures and following description. When this examination is made at an early period, it is found that the young grain of rye is composed of a body or ovary minutely hairy, and of an oval form, (fig. 1, *a*), which is surmounted by a small crown of stiff hairs (*b*), from amidst which the two styles (*c c*) bearing plumose stigmas take their origin from the apex of the grain; at this time the embryo is almost invisible; its place is seen at (*d*); below the grain can be observed the apex of the minute stalk or receptacle (*g*) on which it rests, and from which arise the two scales (*e e*) that cover the base of the ovary; the lines (*f f*) in fig. 1, show the position of the paleæ (*f f*, fig. 3). All of these organs, as well as their position and structure, it is necessary to bear in mind, in order to judge of their alteration in the diseased state.

When the grain of rye is matured, it frequently retains the remains of the stigmas and its hairy crown, as at fig. 2, *b* and *c c*, and always presents an enlarged embryo at its base (*a*), which is joined obliquely to the albumen (*d*) above, and is articulated inferiorly, together with the albumen, to the receptacle (*g*).

When the healthy condition of the young grain was clearly made out, it could easily be seen when that state would be departed from by any particular grain about to become replaced by an ergot; and it is seldom that more than two or three occur on the same spike, as represented in the figure, where the ergot is in its natural position.

The first appearance of the commencement of the growth of the ergot is observed by the young grain and its appendages becoming covered with multitudes of minute cobweb-like filaments, which run over all those parts, cementing anthers and stigmas together (fig 4, *a*), and with a white coating, which appears as if plastered on or left by the evaparation of some liquid, and stuck to the surface of the body of the young ergot, completely concealing it from view, as represented fig. 4, *a*. This white covering could be most easily detached by placing the infected grain in a little water, when countless numbers of minute particles would be loosened from its surface, and ultimately subside to the bottom of the vessel containing the fluid.

On many parts of the spike of the elymus, as well as on the rye, can be observed a viscid fluid, which, according to Philippar, oozes out of the ergot in the stage just described; and the greater the quantity the finer the ergot will be in that particular flower. This liquid is in abundance on the elymus, and in the morning numbers of drops can be collected. I have rather given this an external origin, from the water of dew or rain becoming charged with the particles whilst lodging on the plant;* for my specimens, when cut and placed in water, though they kept alive, exuded no viscid liquid whilst in doors; however, it may arise from the young ergot, as Philippar says, for I have not had many opportunities of watching the increase of this fluid on the growing plant. This fluid is slightly sweet, and contains myriads of the same particles as are deposited on the outside of the ergot. The axis of the ergot, when first appearing, is exceedingly soft; breaking easily across or in any other direction, and exhibiting, in its transverse section, a very irregular lobed or sinuous margin, of a purplish colour, which is surrounded externally by the above-mentioned filaments and particles; this axis appears to be the body of the grain, which has now become changed by the presence and

* It was found that when water is charged with a sufficient quantity of the particles adhering to the ergot, that it becomes viscid and sweetish, and evaporates very slowly; in fact, resembles the fluid that is observed on the exterior of the flowers of ergotized grasses.

growth of the particles and filaments found upon it.

At this early period the size of the ergot is very small, measuring scarcely one-fifth of an inch; still its diminutive condition seems to be most favourable for the support and growth of the particles and filaments upon its surface, (where they increase most rapidly;) for it is found that whilst the ergot is enlarging, there is not a corresponding increase in the number of filaments and particles, but rather a diminution of them, whilst it is advancing to maturity.

In the next stage (fig. 5) we observe the ergot is now grown to show itself just without the paleæ, and begins to show its purplish-black colour, having by this time partially lost its white coating; in fact, when the ergot becomes visible by protruding between the paleæ, the production of filaments and particles has nearly ceased, and the ergot increases in a very rapid manner, according to Philippar only eight or ten days being required to complete its development, attaining in this short period a size four or five times larger than that attained by any healthy grain of the same plant in the same period.

The last stage of the ergot is, that it has elongated much beyond the paleæ that once inclosed it; and puts on a violet-black colour, from the diminution of the filaments and particles that infested it previously. Its length is found to vary in this state from half an inch to one inch and a half, in different specimens. Its form is seldom cylindrical, more frequently obscurely triangular, each side being marked with a furrow, one of them being generally deeper, and more conspicuous than the other two: besides these natural marks, there are a variety of cracks and fissures extending in many different directions. Either end of the ergot is inclined to be pointed, but the lower end more so, and presents a rather smooth extremity or cicatrix, by which it is articulated to the receptacle, between the two scales seen in figs. 4, 5, 6, (*e e*), which are not destroyed by the unnatural growth that springs from between them, and it is extraordinary that Philippar makes no mention of these bodies at the base of the ergot. The summit of the ergot is surmounted (in those specimens which have been carefully gathered) by a small body, which is composed of the remains of the styles, the hairy crown, and a certain portion of the withered grain, as at figs. 5, 6, (*h h*); this body does not exist on the majority of specimens that are procured in the shops, because a very trifling force is sufficient to separate it from the apex of the ergot.

These observations are such as can be easily made with very little microscopic assistance, and have been probably witnesssed by those who have previously paid attention to this subject, and who have given us various opinions respecting its nature; most of which tend to the describing the ergot as a particular fungus, to which we have the different names given by the following botanists, viz. *Sphacelia segetum* by Leveillé; *Sclerotium clavus* by De Candolle; *Clavaria clavus* by Münchhausen; and lastly, *Spermoedia clavus* by Fries, who considers it more analogous to a diseased grain than to any species of fungus.

The contrary to these hitherto received opinions being about to be here advanced, from the results of many examinations of the ergot, in different conditions, and in different grasses, it is fair to explain the reasons for arriving at other conclusions, and those which lead me not to adopt the views of former investigators.

It has been shown that when the young grain of the grass is examined in the healthy state, that its summit bears a tuft of hairs, (particularly evident in *Elymus sabulosus*,) and the two stigmas which spring up amongst them, and at the base of the grain can be observed the two scales, and below the scales is the apex of the pedicle or receptacle, which serves to support the grain, the scales, and the paleæ, seen in fig. 1 and fig. 3. This structure is readily made out in the very young state of the grain, and can also be observed only more or less shrivelled by age, in every condition of the ergot up to its maturity, when the specimens are carefully selected for the purpose, all of which is accurately figured by Phœbus; then as these organs form the appendages at either end of the healthy grain, and they do the same in the ergot, there can be no doubt that the body between these organs in the healthy state is the grain; consequently the body that occupies the same position, but in an altered form, ought to be certainly no other than a grain, which differs from a healthy one, from having in its early state supported a parasite, which communicated to it some disease, which has perverted the normal state of its development.

Notwithstanding the several parts of the grain are arranged as described, Philippar makes out the ergot from his examinations, (which are the best of the later investigators,) to be a separate fungus; still his expressions* are rather vague respecting it; for, speaking of the ergot, he sometimes styles it "ergotized grain made up of fungic substance is the receptacle of the reproductive particles;" in another place, "that the ergot, as a fungus, springs from the receptacular point of the sexual organs; and lastly, he sums up by considering the "ergot as being the reproductive apparatus of a fungus." Philippar's reason for considering it a fungus arises principally from the microscopic examination of the structure of the ergot, which, as a fungus, he describes, beginning in the receptacle of the flower, and lifting up the sexual organs, which are diseased, but still remain upon the apex of the ergot, as in fig. 4; but it is found that where the paleæ are attached, and also the two scales, that this part which must be receptacle also, is not diseased, for these organs remain undisturbed; consequently it can only be the point where the grain and the receptacle unite that could give origin to any body taking the position of the ergot.

[*To be continued.*]

* Vid Traité Organographique, &c., pp. 121, 122, 123.

## Figures and Tables

**Figure f1:**
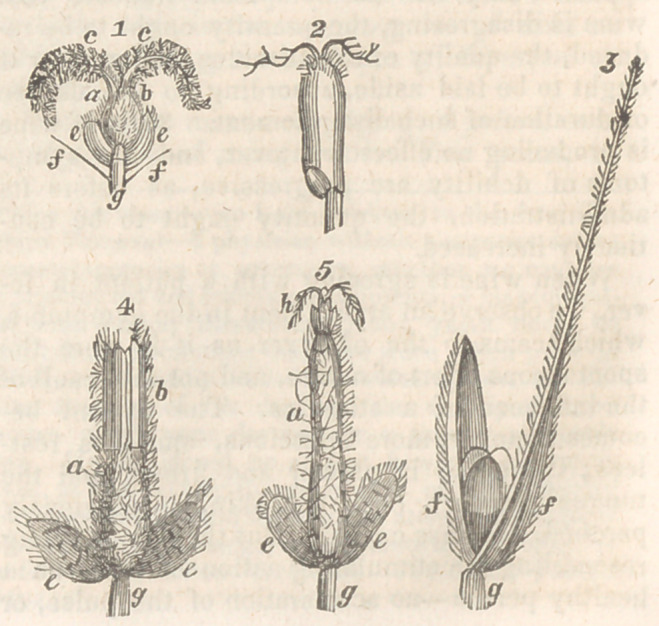


**Figure f2:**